# Impact of COVID-19 on Life Expectancy among Native Americans

**DOI:** 10.1101/2022.03.15.22272448

**Published:** 2022-03-16

**Authors:** Noreen Goldman, Theresa Andrasfay

**Affiliations:** 1 Office of Population Research School of Public and International Affairs Princeton University; 2 Leonard Davis School of Gerontology University of Southern California

## Abstract

**Background:**

There has been little systematic research on the mortality impact of COVID-19 in the Native American population.

**Objective:**

We provide the first estimates of loss of life expectancy directly due to COVID-19 in 2020 and 2021 for the Native American population.

**Methods:**

We use several sources of data (the 2019 life table recently released by the National Center for Health Statistics for Native Americans, provisional COVID-19 deaths by age and race/ethnicity for 2020 and 2021, and population estimates from the US Census Bureau), along with multiple decrement techniques, to calculate life tables for Native Americans that include COVID-19 mortality.

**Results:**

Native Americans had much lower life expectancy than other groups before the pandemic, which set this population behind further: the estimated loss in life expectancy at birth due to COVID-19 for Native Americans is 2.5 years in 2020 and 2.8 years in 2021.

**Conclusions:**

These results underscore the disproportionate share of COVID-19 deaths experienced by Native Americans: a loss in 2020 due to COVID-19 that is almost three times as large as that for Whites and about a half-year greater than that for the Black population. Despite a successful vaccination campaign among Native Americans, the estimated loss in life expectancy at birth due to COVID-19 in 2021 unexpectedly exceeds that in 2020.

**Contribution:**

The increased loss in life expectancy in 2021, despite higher vaccination rates than in other racial/ethnic groups, highlights the huge challenges faced by Native Americans in their efforts to control the deleterious consequences of the pandemic.

## Introduction

Despite heightened media attention to the large number of Native Americans dying from COVID-19, there has been little systematic research on the mortality impact of the coronavirus in this population. One exception is a recent paper demonstrating that age-standardized COVID-19 mortality rates among Native Americans exceeded the corresponding rates for the White, Black and Latino populations during 2020 (Leggat-Barr, Uchikoshi, and Goldman 2021). A separate study based on similar data, in this case for six racial and ethnic groups, indicated that the age-standardized COVID-19 death rate in 2020 among Native Americans was surpassed only by that for Hawaiian and other Pacific Islanders (Feldman and Bassett 2021).

In contrast to the standardized death rate used in these previous papers, measures of reductions in period life expectancy due to COVID-19 quantify the impact of the disease in a way that is easy to interpret while remaining independent of a population’s age distribution. Life expectancy at birth denotes the number of years newborns could expect to live if they experienced the age-specific death rates of a given period throughout their lives; similarly, life expectancy at age 65 reflects the expected number of years of life remaining for a person aged 65, again based on death rates for that period. The impact of COVID-19 on mortality of a population, be it a nation or a subgroup, can meaningfully be assessed in terms of declines in life expectancy between a period prior to and one during the pandemic.

Although the impact of COVID-19 on survival for the White, Black and Latino populations has been assessed in terms of declines in life expectancy (Andrasfay and Goldman 2021a, 2021b; Arias, Betzaida, et al. 2021; Woolf, Masters, and Aron 2021), this useful measure of longevity has not yet been estimated for Native Americans. The major obstacle has been the paucity of high-quality data. Measures of life expectancy are derived from life tables, which, until those published by the National Vital Statistics System (NVSS) in November 2021, had not been available for the total Native American population. Although it would have been possible to use data on published age-specific death rates for Native Americans (e.g., from 2018) to construct a pre-pandemic life table, this life table would not have incorporated the types of corrections used by NVSS to improve data quality.

In this paper, we provide the first estimates of the loss of life expectancy directly due to COVID-19 in 2020 and 2021 for the Native American population. Native Americans, who constitute about two percent of the US population, are defined as those self-identifying in the US Census as American Indian or Alaskan Native (AI/AN), excluding those also identifying as Latino. The analysis by Leggat-Barr and colleagues (2021) suggests that the mortality impact in 2020 was exceptionally large due to the many risk factors for viral exposure and viral severity present in this population: high poverty rates, crowded living arrangements, low access to quality healthcare driven in part by low rates of health insurance (aside from that provided by the poorly funded Indian Health Service (IHS)), frequent employment in frontline jobs, and a high prevalence of comorbidities that increase the risk of COVID-19 fatality. The estimated age-standardized COVID-19 death rate for AI/AN in 2020 exceeded those for other racial/ethnic groups nationally and in most of the states analyzed in their study. In addition, despite the implementation of mitigation strategies by Native American communities, such as contact tracing, sealing borders, mask mandates and enforcement of lockdowns (Foxworth et al. 2021), the state-level COVID-19 death rates in 2020 were strongly correlated with the proportion of Native Americans in the state residing on reservations (Leggat-Barr, Uchikoshi, and Goldman 2021).

To the best of our knowledge, no estimates of the overall mortality impact for Native Americans are currently available for 2021. Despite the high burden of COVID-19 on the AI/AN population in 2020, there are reasons for optimism for 2021. From the start of vaccine availability, the AI/AN population has had higher rates of vaccine uptake than other racial and ethnic groups (Foxworth et al. 2021). Vaccination efforts that were culturally sensitive, combined with a steady supply of vaccine doses, were instrumental to this achievement. Most notable among these strategies was the use of vaccinated community elders as role models who emphasized the importance of preserving AI/AN culture and protecting Native American communities (Foxworth et al. 2021; Silberner 2021).

To assess the impact of COVID-19 on life expectancy in 2020 and 2021, we use the newly published 2019 life table for the Native American population to provide information on death rates and life expectancy just prior to the pandemic (Arias, Xu, et al. 2021). These data have been adjusted by NVSS via linkages between the 2010 Census and death registration data to correct for various types of misreporting, particularly misclassification of race and ethnicity on death certificates, a well-recognized and serious source of bias in data for the Native American population that has resulted in underestimation of mortality (Arias, Xu, et al. 2021; Espey et al. 2014; Jim et al. 2014). We then use data on provisional COVID-19 deaths and population estimates for Native Americans to estimate the loss in life expectancy at birth and at age 65 between 2019 and 2020 and between 2019 and 2021 due to COVID-19. These estimates, which are based on multiple decrement techniques described below, are compared with corresponding values of life expectancy decline for the White, Black, and Latino populations in 2020.

## Data and Methods

Data on provisional COVID-19 deaths by age, race, and ethnicity in 2020 and 2021 are obtained from the National Center for Health Statistics and were last updated March 2, 2022 (NCHS 2022). The 2019 life table for the AI/AN population was provided by the NVSS (Arias, Xu, et al. 2021), and the mid-year estimates of population size for 2020 and 2021 were obtained from the US Census Bureau (US Census Bureau 2021).

Because all-cause mortality data by race and ethnicity are not yet available for 2021 and typically do not become available for at least a year beyond the reference year, we calculate life tables during the pandemic using multiple decrement life table methods, which were designed to estimate the impact of eliminating a cause of death (Chiang 1968). We treat the 2019 life tables as mortality conditions that would be expected if COVID-19 were eliminated as a cause of death and quantify how the inclusion of COVID-19 deaths affected life expectancy. Since these calculations incorporate only COVID-19 deaths, not other excess deaths, we refer to our estimates as reductions (directly) due to COVID-19, rather than the total reductions expected in 2020 and 2021. Additional details on this method are available elsewhere (Andrasfay and Goldman 2021b).

## Results

There were 4,615 COVID-19 deaths to Native Americans in 2020 and 4,995 in 2021. The median ages of these deaths declined from 69 to 65 years over this period, which, combined with the increase in numbers of deaths, suggests, contrary to expectation, a more severe decline in life expectancy at birth in 2021.

**Figure 1:**
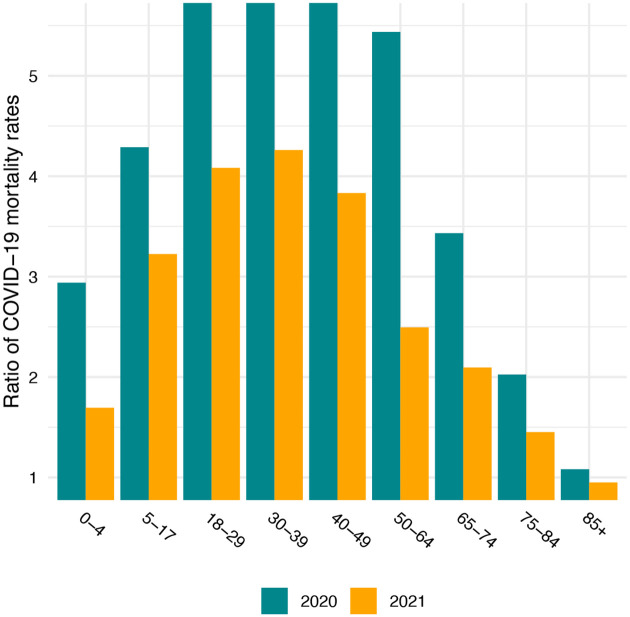
Ratio of Native American to White COVID-19 age-specific mortality rates in 2020 and 2021.

Brought about partly by the younger age distribution of the Native American than the White population (Akee and Reber 2021; Leggat-Barr, Uchikoshi, and Goldman 2021), the median ages of COVID-19 deaths are more than a decade higher for Whites – 82 in 2020 and 75 in 2021 – than for Native Americans. However, as shown in Figure 1, these differences in ages at death primarily reflect the much higher COVID-19 mortality rates among Native Americans at younger ages. COVID-19 death rates in the young adult and middle age range are about five times as high for Native Americans as for Whites in 2020, modestly reduced to about four times as high in 2021. In contrast, above age 65, death rates are less than twice as high among Native Americans. Preliminary work suggests that the Black and Latino populations also experienced reductions in age-specific death rates relative to Whites from 2020 to 2021 driven by rising mortality rates among Whites (Andrasfay and Goldman 2022).

Table 1 presents life expectancy at birth (e_0_) and at age 65 (e_65_) as calculated by NCHS in their 2019 life tables and as estimated here for 2020 and 2021 based on the multiple decrement calculation described above. The estimated declines in life expectancy at birth due only to COVID-19 are 2.5 years in 2020 and a larger estimate of 2.8 years in 2021. The effect of introducing COVID-19 mortality is to reduce Native American life expectancy at birth during the pandemic to just 69 years. This is substantially below the 71.1-year e_0_ calculated for this population in 2007–2009, an estimate based on only 64% of the AI/AN population, i.e., those residing in the 637 IHS Contract Health Service Delivery Areas (Arias, Xu, et al. 2021; Arias, Xu, and Jim 2014). In contrast to the results for e_0_, the estimated impact of COVID-19 on e_65_ declined from 2020 to 2021, by 0.2 years.

**Figure 2:**
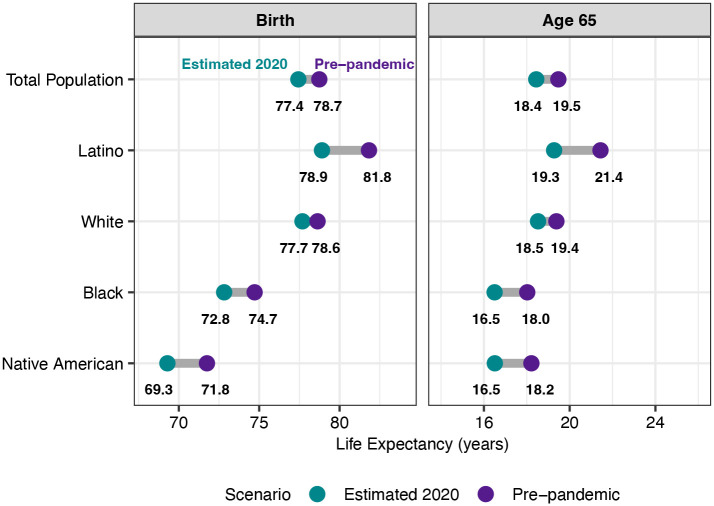
Life expectancy at birth and at age 65 by race/ethnicity, estimates for 2020 compared to pre-pandemic. Due to differences in data availability, pre-pandemic refers to 2019 for the Native American population and 2018 for the other populations. Estimates for the Total, Latino, White, and Black populations are authors’ calculations using the same methods as for the Native American population.

Figure 2 compares the estimated loss in life expectancy due to COVID-19 in 2020 across racial and ethnic groups, using estimates with the same cause-deleted life table methods for all racial/ethnic groups. This figure shows that, even before the pandemic, the Native American population had much lower life expectancy than other major racial/ethnic groups in the US. The pre-pandemic (2019) e_0_ of 71.8 for the AI/AN population was nearly seven years below that of the White population and a decade lower than that of the Latino population. Deaths from COVID-19 have set this population behind even further: the loss in e_0_ for Native Americans is estimated to be almost three times as large as for Whites and to exceed that for the Black population by about a half-year. The estimated e_0_ reduction falls below that for Latinos by about a half-year, but these losses represent similar proportionate reductions for both populations. The 1.7-year loss in e_65_ for Native Americans is similar to that for the Black population but below that for Latinos.

## Discussion

Throughout the pandemic, scholars and journalists have described the disproportionate share of COVID-19 deaths experienced by Native Americans. Here, we quantified this mortality burden in terms of reductions in life expectancy at birth and at age 65. We estimate that COVID-19 deaths have reduced life expectancy at birth in 2020 and 2021 to approximately 69, an astonishingly low value in a high-income country. This level of life expectancy is below that in every country in the Americas with the sole exception of Haiti, and similar to values in India, Pakistan and Nepal (Population Reference Bureau 2020). COVID-19 deaths have exacerbated an already stark mortality disparity between Whites and Native Americans in the US: our estimates suggest that COVID-19-associated reductions in 2020 life expectancy at birth were nearly three times as large for Native Americans as they were for Whites.

The situation is even more egregious than these results suggest because our analysis almost certainly underestimates total life expectancy loss during the pandemic, for two main reasons. One is that, in contrast to the death information used to construct the 2019 life table for Native Americans, the numbers of COVID-19 deaths in 2020 and 2021 have not been adjusted for potential undercounts of deaths among AI/AN individuals or delayed reports of COVID-19 deaths, which are especially likely to affect counts for 2021. Earlier studies indicate that at least 30 percent of individuals who self-identify as non-Latino AI/AN in US Censuses or the Current Population Survey are classified differently on death certificates, leading to a large underestimate of death rates (Arias, Xu, et al. 2021). Underreporting of Native American race on death certificates has been especially prevalent for persons self-identifying with multiple races, a group often residing outside areas designated for Native Americans (Jim et al. 2014). A second reason is that our estimates are designed to examine the impact of only direct COVID-19 mortality and do not account for increases in death rates from other causes that may have been brought about indirectly by broader consequences of the pandemic or by elevated mortality among COVID-19 survivors (Ackley et al. 2022; Al-Aly, Xie, and Bowe 2021). Given the particularly high rates of chronic disease and COVID-19 infection in the AI/AN population, along with population-wide increases in detrimental health-related behaviors and reduced availability and use of healthcare during the pandemic, non-COVID-19 mortality among Native Americans probably increased substantially after 2019 (Hatcher et al. 2020; Leggat-Barr, Uchikoshi, and Goldman 2021). There is already evidence of a more than 40% increase in fatal drug overdoses among Native Americans in 2020, which exceeds the increases for Whites and Latinos (Friedman and Hansen 2022; Hedegaard et al. 2021).

Despite the successful vaccination campaign among Native Americans, as well as efforts to increase COVID-19 testing and modernize the health IT infrastructure of the IHS (Indian Health Service 2022), the estimated loss in life expectancy at birth due to COVID-19 in 2021 exceeds that in 2020. There are several plausible explanations for this unexpected and disturbing finding. First, because the initial vaccine rollout prioritized healthcare workers, no racial/ethnic group had substantial vaccination coverage during January-February 2021 – two of the deadliest months of the pandemic (Centers for Disease Control and Prevention 2022; Ndugga et al. 2021). Second, two highly contagious variants that partially evaded vaccine-acquired and natural immunity, Delta and Omicron, emerged in 2021 (Kupferschmidt and Wadman 2021; del Rio, Omer, and Malani 2021). Third, the Native American population, like the general US population, still has a substantial proportion of unvaccinated persons, especially among younger adults, and a relatively low uptake of booster doses (Centers for Disease Control and Prevention 2022). Fourth, and most importantly, Native Americans continue to experience large social, economic and health inequities, some of which have persisted for centuries. These factors increase risks of COVID-19 infection, hospitalization and death: high rates of poverty, poor housing infrastructure including inadequate plumbing, crowded living conditions often involving multigenerational families, employment in low-income frontline jobs that cannot be performed remotely, high rates of co-morbidities (particularly obesity and diabetes) that increase the severity and fatality of COVID-19, a high prevalence of smoking, low rates of health insurance other than the IHS, and poorly resourced healthcare systems that provide inadequate and often inaccessible care (Leggat-Barr, Uchikoshi, and Goldman 2021). Because many of these risk factors are more prevalent in tribal lands than in non-tribal areas, vulnerability to COVID-19 is especially high for Native Americans residing on reservations (Leggat-Barr, Uchikoshi, and Goldman 2021).

Although COVID-19 death rates among Native Americans undoubtedly would have been larger in the absence of a successful vaccine campaign, our results underscore the huge challenges faced by this population in their efforts to control the appalling consequences of the ongoing pandemic as well as continued high rates of morbidity and mortality from other causes. The large financial investment in the American Rescue Plan to enhance identification and treatment of COVID-19 infections and strengthen the public health infrastructure for the Native American population is a significant step forward (Indian Health Service 2021).

## Figures and Tables

**Table 1:** Life expectancy estimates and reductions due to COVID-19 for the Native American population

	Birth (e_0_)	Age 65 (e_65_)
**2019**		
Life expectancy	71.8	18.2
**2020**		
COVID-19 deaths among Native Americans	4,615	
Life expectancy	69.3	16.5
Reduction in life expectancy due to COVID-19	2.5	1.7
**2021**		
COVID-19 deaths among Native Americans	4,995	
Life expectancy	69.0	16.7
Reduction in life expectancy due to COVID-19	2.8	1.5

Notes: Apart from life expectancy values from 2019 that are provided by the National Vital Statistics System, all life expectancy estimates are authors’ calculations. Estimates for 2020 and 2021 estimates are based on provisional COVID-19 death counts provided by the National Center for Health Statistics (March 2, 2022 update).
